# Supplementary material for: Poly(oxyethylene)/Poly(oxypropylene) butyl ether prolongs the repellent effect of N,N-diethyl-3-toluamide on the skin
Source: PLoS One. 2023 Oct 3;18(10):e0292447. doi: 10.1371/journal.pone.0292447 (PMC10547171; doi:10.1371/journal.pone.0292447)
Supplement: Attachment — Submitted filename: 230825Response_to_Reviewers.docx [file pone.0292447.s001.docx]

**Response to Reviewer Comments**

We carefully revised our manuscript according to the suggestions of the reviewers, and details are as follows.

**< Q and A for Reviewer 1>**

**Q1. In the drug efficacy evaluation experiment (in vivo), DEET-E results were stopped in the middle of the study. Author should add the reason for this.**

A1. Thank you very much for pointing this out. It is known that a minimum of 90% repellent rate is required for effective repellency. Taken together, the endpoint of the measurement was set at the point when the repellent rate showed below 90%. In order to respond to the reviewer’s comment, we added the information in the Materials and methods (line 223-225).

**Q2. Why different temperatures were used in viscosity and degradability of DEET formulation?**

A2. The reviewer’s comment is correct. The temperature was affected the viscosity and degradability of DEET. Therefore, we measured the changes in viscosity and degradability of DEET under the temperature range in daily life (4 °C, 25 °C, and 50 °C). In order to respond to the reviewer’s comment, we added the content in the Results (line 242-245).

**Q3. Why did you add ethanol to the formula. Author should indicate whether commercial DEET products also contain 40% ethanol.**

A3. It is mainly dispersed in 40% ethanol, since DEET does not have a high affinity for water. From these background, many commercial DEET products also use 40% ethanol as a solvent. In order to respond to the reviewer’s comment, we added the information in the Discussion (line 339).

**Q4. Figure 2B, is there any reason to check the degradability after one month? Have you checked it at different days or week? What happens before and after one month? There should be any rationale to point out one specific time point.**

A4. The reviewer’s comments are very important. In this study, we collected the samples every 1, 2, 3, and 4 weeks, and measured changes over time. The concentration of DEET was similar before and after one month. In order to respond to the reviewer’s comment, we added these contents in the Materials and methods, and Discussion (line 163, 341-342).

**Q5. Tensile stress is thought to be affected by temperature. How high was the temperature when measuring tensile stress?**

A5. Thank you very much for pointing this out. The measurement of tensile stress was performed at 22 °C. In order to respond to the reviewer’s comment, we added this information in the Materials and methods (line 115).

**Q6. In evaluating repellent effect of DEET, how large a space were female A. albopictus released into? Author should add this information.**

A6. The reviewer’s comment is correct. The evaluation of repellent effect of DEET was performed in 25×25×25 cm metal mesh cages (50 female mosquitoes per cage). In order to respond to the reviewer’s comment, we added this information in the Materials and methods (line 212-213).

Thank you for great comments.

**< Q and A for Reviewer 2>**

**Q1. Please clarify which humane endpoints were established in your study.**

A1. Thank you very much for pointing this out. The death of a regulated animal as a likely outcome or planned experimental endpoint is not included in this study. Thank you for pointing out this.

**Q2. Please provide ethical guidelines to justify the types and doses of anesthetic agents used for anesthesia and euthanasia of rat in your study.**

A2. Thank you for pointing out this. The types and doses of anesthetic agents used for anesthesia and euthanasia of mice and rats were determined according to AVMA Guidelines for the Euthanasia of Animals: 2020 Edition. In order to respond to the editor’s comment, we added the information in the in the Materials and methods (line 97-99).

**Q3. Please indicate how your results compare with those from previous studies using cyclodextrin on this topic published in the literature.**

A3. The reviewer’s comments are very important. The DEET formulation containing POE-POP was less tensile stress, and prolonged the retention of DEET in the superficial layers of the rat skin in comparison with DEET formulation containing cyclodextrins. From these results, the addition of POE-POP is expected to lead to the development of good DEET formulations. In order to respond to the editor’s comment, we added the information in the in the Conclusions (line 416-418).

**Q4. The addition of POE-POPs increases hydrophilicity. Please explain more clearly why increased hydrophilicity would decrease skin penetration of DEET.**

A4. Thank you very much for pointing this out. Skin surfaces such as stratum corneum are hydrophobic, and the enhanced hydrophilicity of DEEF formulations attenuated their ability to penetrate into the skin. In order to respond to the reviewer’s comment, we added the information in the in the Discussion (line 366-368).

**Q5. Addition of POE-POP formed the micelles. Is there any change in color? Please add photos of the formulation with and without POE-POP.**

A5. The reviewer’s comment is correct. No change in color tone was observed regardless of addition or non-addition of POE-POP. In order to respond to the reviewer’s comment, we added photos of the formulation with and without POE-POP in the Figure 1E (Figure 1E).

Thank you for great comments.
